# lncRNA H19 is involved in TGF-*β*1-induced epithelial to mesenchymal transition in bovine epithelial cells through PI3K/AKT Signaling Pathway

**DOI:** 10.7717/peerj.3950

**Published:** 2017-10-17

**Authors:** Wei Yang, Xuezhong Li, Shaopei Qi, Xueru Li, Kun Zhou, Suzhu Qing, Yong Zhang, Ming-Qing Gao

**Affiliations:** 1 College of Veterinary Medicine, Northwest A&F University, Yangling, Shaanxi, China; 2 Innovation Experimental College, Northwest A&F University, Yangling, Shaanxi, China; 3 Key Laboratory of Animal Biotechnology, Ministry of Agriculture, Northwest A&F University, Yangling, Shaanxi, China

**Keywords:** Mastitis, Epithelial cells, MAC-T, H19, TGF-β1, Bovine

## Abstract

Increased levels of long noncoding RNA H19 (H19) have been observed in many inflammatory and organ fibrosis diseases including ulcerative colitis, osteoarthritis, liver fibrosis, renal fibrosis and pulmonary fibrosis. However, the role of H19 in bovine mastitis and mastitis-caused fibrosis is still unclear. In our study, H19 was characterized as a novel regulator of EMT induced by transforming growth factor-β1 (TGF-β1) in bovine mammary alveolar cell-T (MAC-T) cell line. We found that H19 was highly expressed in bovine mastitis tissues and inflammatory MAC-T cells induced by virulence factors of pathogens. TGF-β1 was also highly expressed in inflammatory MAC-T cells, and exogenous TGF-β1 could induce EMT, enhance extracellular matrix protein expression, and upregulate H19 expression in epithelial cells. Stable expression of H19 significantly promotes EMT progression and expression of ECM protein induced by TGF-β1 in MAC-T cells. Furthermore, by using a specific inhibitor of the PI3K/AKT pathway, we demonstrated that TGF-β1 upregulated H19 expression through PI3K/AKT pathway. All these observations imply that the lncRNA H19 modulated TGF-β1-induced epithelial to mesenchymal transition in bovine epithelial cells through PI3K/AKT signaling pathway, which suggests that mammary epithelial cells might be one source for myofibroblasts *in vivo* in the mammary glands under an inflammatory condition, thereby contributing to mammary gland fibrosis.

## Introduction

Bovine mastitis is a common disease that occurs in dairy herds and is caused by changes in metabolism, physiological trauma, and contagious or environmental pathogenic microorganisms (Oviedo-Boyso et al., 2007). Mammary tissue is damaged by products of bacterial pathogens and enzymes released from stroma cells and secretory cells during an immune response if infection persists (Zhao & Lacasse, 2008). The repair process in damaged mammary tissue is typically accomplished by fibrosis, which can begin during an inflammatory response (Benites et al., 2002). Both mastitis and mastitis-caused fibrosis affect the dairy industry by reducing milk production and increasing treatment costs.

Epithelial mesenchymal transition (EMT) is the process by which epithelial cells gradually acquire certain characteristics of mesenchymal cells to produce fibroblasts and myofibroblasts (He et al., 2017). It is characterized by morphological changes in epithelial cells from a cobblestone-shape to a spindle-shape and expression changes in some EMT markers, such as decreased E-cadherin and increased vimentin and α-smooth muscle actin (α-SMA) (Thiery, 2002). An increasing number of studies has shown that EMT plays a role in the genesis of fibroblasts during organ fibrosis (Becker-Carus, 1972). Additionally, transforming growth factor (TGF)-β1 can induce EMT during organ fibrosis and is regarded as a master regulator of EMT progression (Moustakas & Heldin, 2016; Risolino et al., 2014).

Long noncoding RNAs (lncRNAs) are a class of transcripts longer than 200 nucleotides, and the majority do not have protein coding potential (Kwok & Tay, 2017). Presently, numerous functional lncRNAs have been characterized and recognized as critical regulators of various physiological and pathological processes (Engreitz, Ollikainen & Guttman, 2016). Although many lncRNAs have been reported to modulate cancer (Yuan et al., 2014) cardiovascular diseases (Archer et al., 2015), neurological disorders (Briggs et al., 2015) and inflammation (Mathy & Chen, 2017; Zacharopoulou et al., 2017), the specific roles of lncRNAs in mediating the role of TGF-β1 and regulating EMT in bovine epithelial cells during mastitis-caused fibrosis process are not well studied. LncRNA H19 (H19) gene is an imprinted maternally expressed gene located on chromosome 29 in bovine that plays a vital role in mammalian development (Keniry et al., 2012). Increasing evidence has shown that H19 may have either oncogenic or tumor suppressor properties based on its opposite expression changes in various cancers, including bladder cancer (Luo et al., 2013) and gastric cancer (Song et al., 2013; Yang et al., 2012), but the exact mechanism is unclear (Jiang et al., 2016). H19 RNA is related to the development of inflammatory diseases such as ulcerative colitis (Chen et al., 2016) and osteoarthritis (Steck et al., 2012), as well as organ fibrosis including liver fibrosis (Song et al., 2017), renal fibrosis (Xie et al., 2016), and pulmonary fibrosis (Tang et al., 2016). A recent study linked H19 upregulation to the TGF β-induced EMT process (Matouk et al., 2013).

In this study, we compared the expression of H19 in inflammatory tissue and mammary epithelial cells to that in normal cells. Using the immortalized mammary epithelial cell line MAC-T, we investigated the roles of H19 in TGF-β1-induced EMT in MAC-T cells. The results suggest that H19 mediates mastitis-caused mammary fibrosis. This finding improves the understanding of the pathological mechanism of bovine mammary gland fibrosis caused by mastitis.

## Materials and Methods

### Cell culture and treatment

The MAC-T cell line was a gift from Prof. Mark D. Hanigan (Virginia Polytechnic Institute and State University, Blacksburg, VA, USA). MAC-T cells were cultured in complete DMEM/F12 medium (GIBCO BRL, Life Technologies, Grand Island, NY, USA) supplemented with 10% fetal bovine serum, 100 IU/mL penicillin, and 100 µg/mL streptomycin (Gibco BRL) at 37 °C in an incubator with 5% CO_2_. Confluent cells were subcultured by digestion with 0.15% trypsin and 0.02% EDTA.

For cell treatments with reagents, cells grown to approximately 80% confluence in culture dishes were respectively treated with lipopolysaccharide (LPS) (Sigma-Aldrich, St. Louis, MO, USA) at 10 ng/µL for 3 h, lipoteichoic acid (LTA) (Inviogen, Carlsbad, CA, USA) at 20 ng/µL for 12 h, or TGF-β1 (Creative BioMart, Shirley, NY, USA) at 10 ng/mL for 36 h according to recommended concentrations in our previous publication (Zhang et al., 2016).

### RNA extraction and real-time PCR

Total mRNA was extracted from mammary tissue and cells by using TriZol solution (TransGene, Shanghai, China) and the RNA Easy Kit (TransGene) according to the manufacturer’s instructions. Total RNA concentration was measured by a spectrophotometer (ND 2.0; Nano Drop Technologies, Wilmington, DE, USA), and 3 µg of total mRNA was reverse-transcribed into cDNA using TransScript II First-Strand cDNA Synthesis SuperMix (TransGene). Quantitative primers were designed based on the sequences in the National Center of Biotechnology Information Database and synthesized by Sangon Biotech (Shanghai, China). The primers are listed in File S1. Quantitative real-time was performed with an iQ5 light cycler (Bio-Rad, Hercules, CA, USA) in 20-µL reactions. GAPDH was used as a reference gene.

### Western blotting

Cells were lysed in PRO-PREP Protein Extraction Solution (iNtRON Biotechnology, Inc., Gyeonggi-do, Korea) to prepare protein lysates according to the manufacturer’s instructions. Cell lysates were centrifuged at 12,000 rpm for 10 min at 4 °C. Protein concentration was measured using Bradford Easy Protein Quantitative Kit (TransGene). Equal amounts of protein were separated in 10% polyacrylamide gels (Sigma-Aldrich) and transferred to polyvinylidene fluoride membranes. The membranes were blocked with 10% non-fat milk and incubated with anti-α-SMA (Abcam, Cambridge, UK), anti-collagen I (Abcam), anti-E-cadherin (Abcam), anti-N-cadherin (Bioss, Beijing, China), albumin (Bioss), matrix metalloproteinase 9 (MMP9) (Bioss), vimentin (Bioss), pan-cytokeratin (a wide spectrum cytokeratin antibody, Bioss), fibronectin (Bioss), β-catenin (Sigma) and anti-GAPDH (TransGene) antibodies at 4 °C overnight. After washing the membranes with Tris-buffered saline containing Tween 20 three times for 5 min each time, the membranes were incubated with secondary antibodies. Finally, immunoreactive proteins were detected using an enhanced chemiluminescence detection kit (Beyotime, Shanghai, China).

### Enzyme-linked immunosorbent assay (ELISA)

MAC-T cells were cultured for 24 h in fresh serum-free medium after treatment with LPS or LTA. The medium was collected and centrifuged at 12,000 rpm for 5 min to remove cell debris. Tumor necrosis factor α (TNF-α) secreted from MAC-T in the medium was detected according to the ELISA kit instructions (Huzhen Biological Technology Co., Ltd., Shanghai, China).

### Establishment of stable MAC-T cell clones overexpressing H19

The full-length H19 gene sequence from NCBI was synthesized by Sangon Biotech and contained BamHI and NotI enzyme restriction sites. The synthesized product was digested with the BamHI and NotI enzymes and ligated into the BamHI and NotI sites of the CD513B-1 basic vector (System Biosciences, Mountain View, CA, USA), yielding the CD513B-1-H19 construct. One day before transfection, MAC-T cells were seeded into 60-mm culture dishes at approximately 80% confluence. Cells were transfected with CD513B-1-H19 or CD513B-1 control plasmids by electroporation with transfer buffer in a 4-mm gap cuvette at 510 V for one pulse. After 8 h of transfection, cells were subcultured to 50% confluence in medium containing 0.6 µg/mL puromycin (Sigma-Aldrich). When all MAC-T cells in the non-transfected control culture were killed, puromycin-resistant clones were picked and passaged in medium containing half the concentration of puromycin. H19-targeted cell clones were screened by real-time PCR and flag fluorescence.

### Luciferase reporter assays

A series of upstream fragments of the H19 gene sequence were amplified by PCR from genomic DNA using primers containing enzyme restriction sites; the primers are listed in File S1. These fragments of the H19 gene sequence were respectively cloned into the PGL4.10 plasmids (Promega, Madison, WI, USA). MAC-T cells were transfected with a mixture of pRL-TK-renilla-luciferase plasmid and PGL4.10-reporter plasmids. The PGL4.10 plasmid was used as a control vector. After transfection for 8 h, MAC-T cells transfected with PGL4.10-reporter plasmids and control vectors were treated with TGF-β1 as described above. Finally, luciferase activities were detected by a Dual-Luciferase Reporter analytical instrument (Promega).

### Statistical analysis

The results are expressed as the means  ± standard deviation (SD). All data from at least three independent experiments for each parameter were analyzed by analysis of variance (SPSS11.5 software, SPSS, Inc., Chicago, IL, USA). A *p*-value of <0.05 was considered statistically significant.

## Results

### Expression of H19 in mammary tissue and epithelial cells of bovine

**Figure 1:**
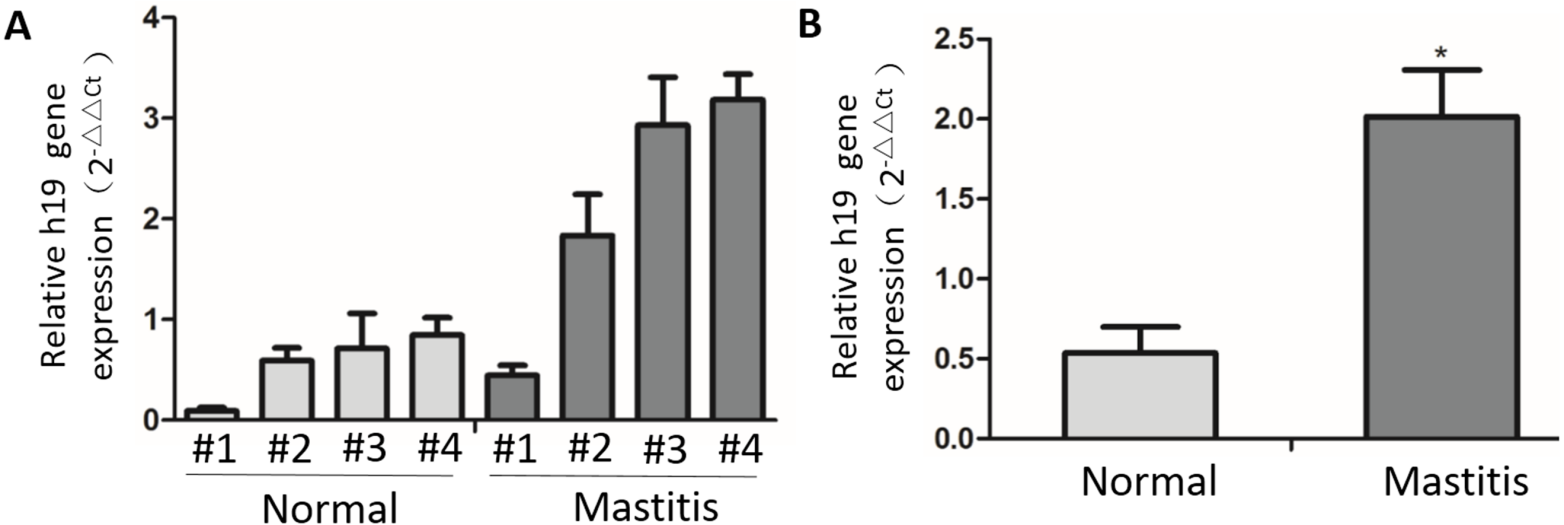
The expression of H19 in normal and mastitic tissue of bovine mammary glands. (A) Expression of the H19 gene in normal (*n* = 4) and mastitic (*n* = 4) tissue of mammary glands was assessed by RT-qPCR. GAPDH was used as an internal control. (B) Statistical analysis of the expression of H19 gene between normal and mastitis group. ^∗^*p* < 0.01 vs normal.

**Figure 2:**
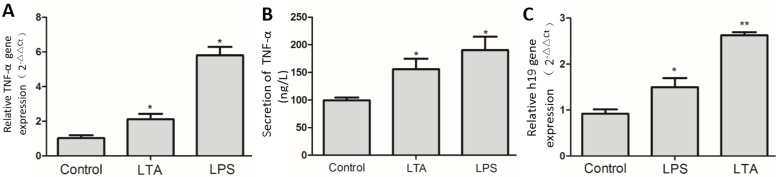
Expression of H19 in MAC-T cells with or without LPS or LTA treatment. (A and B) An inflammatory epithelial cell model was established by LPS or LTA stimulation *in vitro*. TNF-α was used as an indicator of MAC-T cell inflammatory responding to stimulus. TNF-α mRNA expression in treated cells was analyzed by RT-qPCR (A), and TNF-α protein secretion in the medium was measured with an ELISA kit (B). (C) Expression of H19 in MAC-T cells was analyzed by RT-qPCR. Untreated MAC-T cells were used as a control. GAPDH was used as an internal control. ^∗^*p* < 0.05, ^∗∗^*p* < 0.01 vs control.

To investigate whether H19 mediates the process of bovine mastitis, we first examined the expression of H19 in normal and inflammatory bovine mammary tissue. Results from qPCR analysis showed that H19 was significantly up-regulated in the inflammatory mammary tissue compared to normal tissue (Fig. 1). We further detected H19 expression in inflammatory MAC-T cells in an epithelial cell model of mastitis by treating MAC-T cells with LPS or LTA according to the concentration recommended in our previous publication (Zhang et al., 2016). Both LPS and LTA induced an obvious inflammatory response in MAC-T cells as indicated by elevated TNF-α mRNA expression (Fig. 2A) and protein secretion (Fig. 2B). Consistent with the expression of H19 at the tissue level, H19 expression was also up-regulated in inflammatory epithelial cells induced by LPS and LTA *in vitro* (Fig. 2C).

### TGF-*β*1 was highly expressed in inflammatory epithelial cells and induced EMT and enhanced extracellular matrix (ECM) protein expression

**Figure 3:**
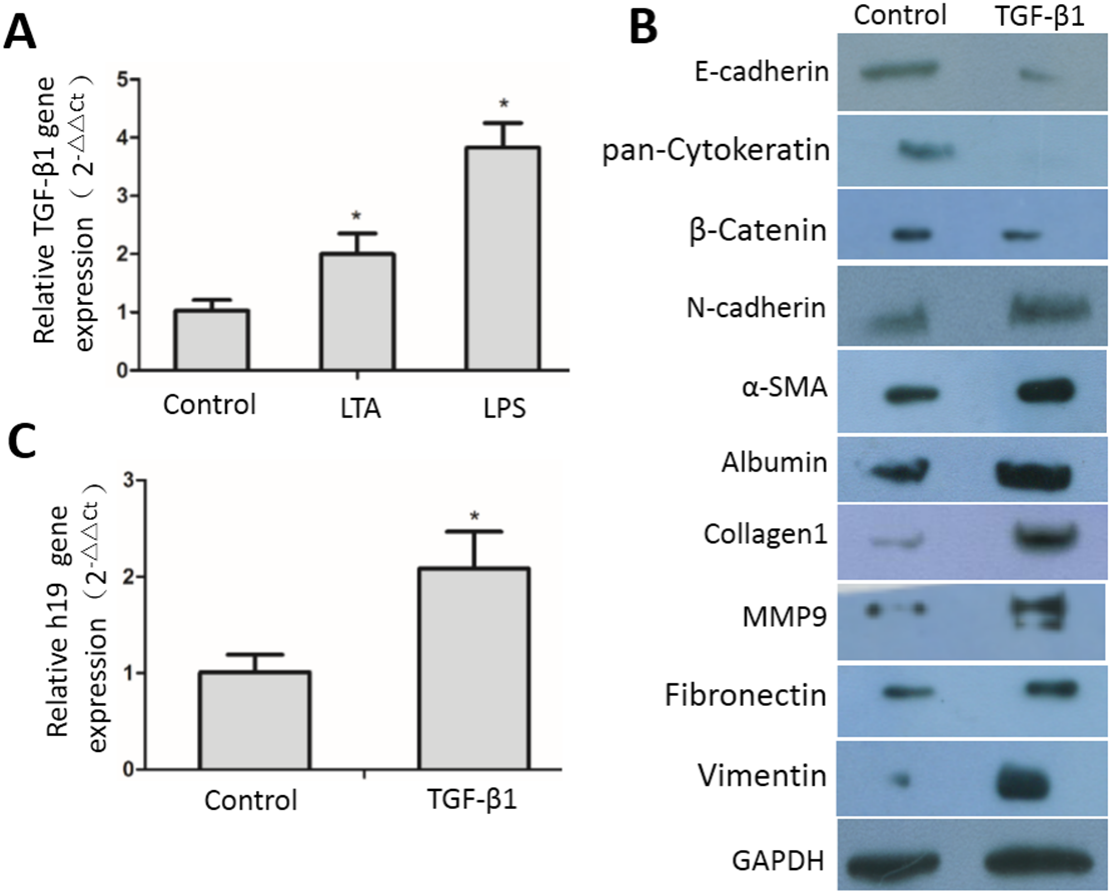
TGF-β1-induced expression of EMT markers, ECM proteins and H19 in MAC-T cells. (A) Expression of TGF-β1 gene in inflammatory MAC-T cells was analyzed by RT-qPCR. (B) Expression of EMT markers (E-cadherin, N-cadherin, pan-cytokeratins, vimentin, β-catenin and α-SMA), ECM protein (albumin, MMP9, collagen type I and fibronectin ) in MAC-T cells were analyzed by western blotting. (C) Expression of H19 in MAC-T cells upon TGF-β1 treatments was analyzed by RT-qPCR. Untreated MAC-T cells were used as a control. GAPDH was used as an internal control. ^∗^*p* < 0.01 vs control.

TGF-β1 has been shown to play an essential role in suppressing inflammation, but recent studies have revealed positive roles of TGF-β1 in the inflammatory response (Yoshimura, Wakabayashi & Mori, 2010). Here we found that TGF-β1 expression was increased in both LPS- and LTA-induced inflammatory MAC-T cells (Fig. 3A). TGF-β1 induces EMT phenotypes in epithelial cells *in vitro* and has been associated with EMT *in vivo* (Moustakas & Heldin, 2016; Risolino et al., 2014). To investigate whether increased TGF-β1 in epithelial cells under inflammatory conditions caused mammary fibrosis via EMT, we incubated MAC-T cells with exogenous TGF-β1 to examine the occurrence of EMT. The western blot results showed that the expression of α-SMA, N-cadherin and vimentin, three well-known EMT markers, was significantly upregulated in TGF-β1-treated MAC-T cells compared to that in untreated cells, while the expression of E-cadherin, β-catenin and pan-cytokeratin was down-regulated in MAC-T cells after treatment with TGF-β1 compared to that in untreated cells (Fig. 3B), suggesting that TGF-β1 induces EMT in MAC-T epithelial cells. In addition, TGF-β1 treatment enhanced the protein expression levels of several ECM proteins, including albumin, fibronectin, collagen 1, and MMP9 in MAC-T cells (Fig. 3B). Interestingly, we found that TGF-β1 treatment up-regulated H19 expression in MAC-T cells (Fig. 3C).

**Figure 4:**
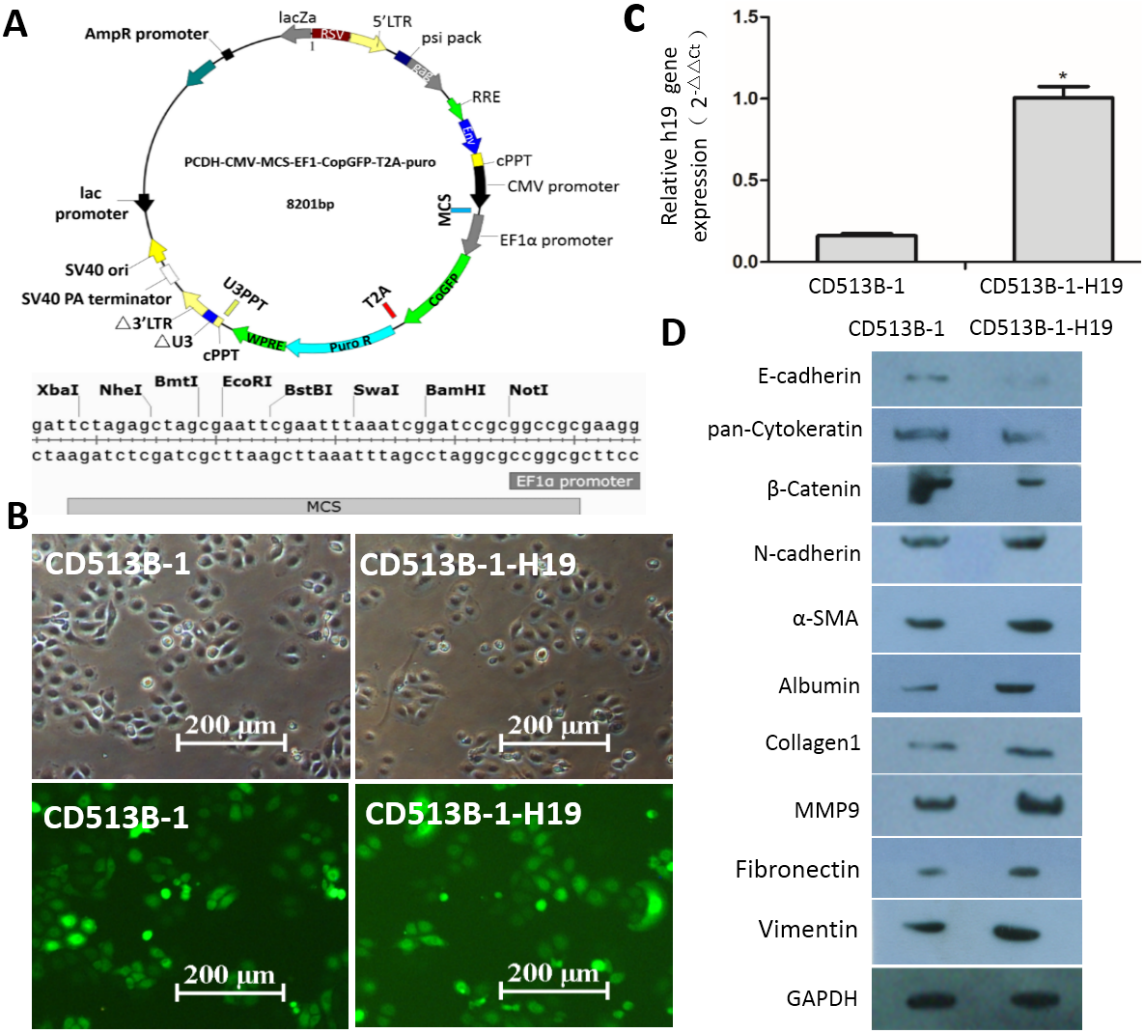
H19 promotes EMT progression and ECM protein expression induced by TGF-β1 in MAC-T cells. (A) Information for the lentviral vector CD513B-1. BamHI and NotI sites in CD513B-1 were used to construct the expression plasmid. (B) Fluorescence microscopy examination of MAC-T cells transfected with lentviral vectors. GFP expression was observed by fluorescence microscopy. Up, MAC-T cells infected with CD513B-1 or CD513B-1-H19 were detected in bright field; down, MAC-T cells infected with CD513B-1 or CD513B-1-H19 were detected in dark field. (C) Analysis of H19 expression in MAC-T cells with empty vector or CD513B-1-H19 by RT-qPCR. (D) The expression of EMT marker proteins (E-cadherin, N-cadherin, pan-cytokeratin, β-catenin, vimentin and α-SMA) and ECM proteins (albumin, collagen 1, fibronectin and MMP9) were evaluated in H19-overexpressing MAC-T cells by western blotting. Control vector-transduced cells were used as a control. GAPDH was used as an internal control. ^∗^*p* < 0.05 vs control.

**Figure 5:**
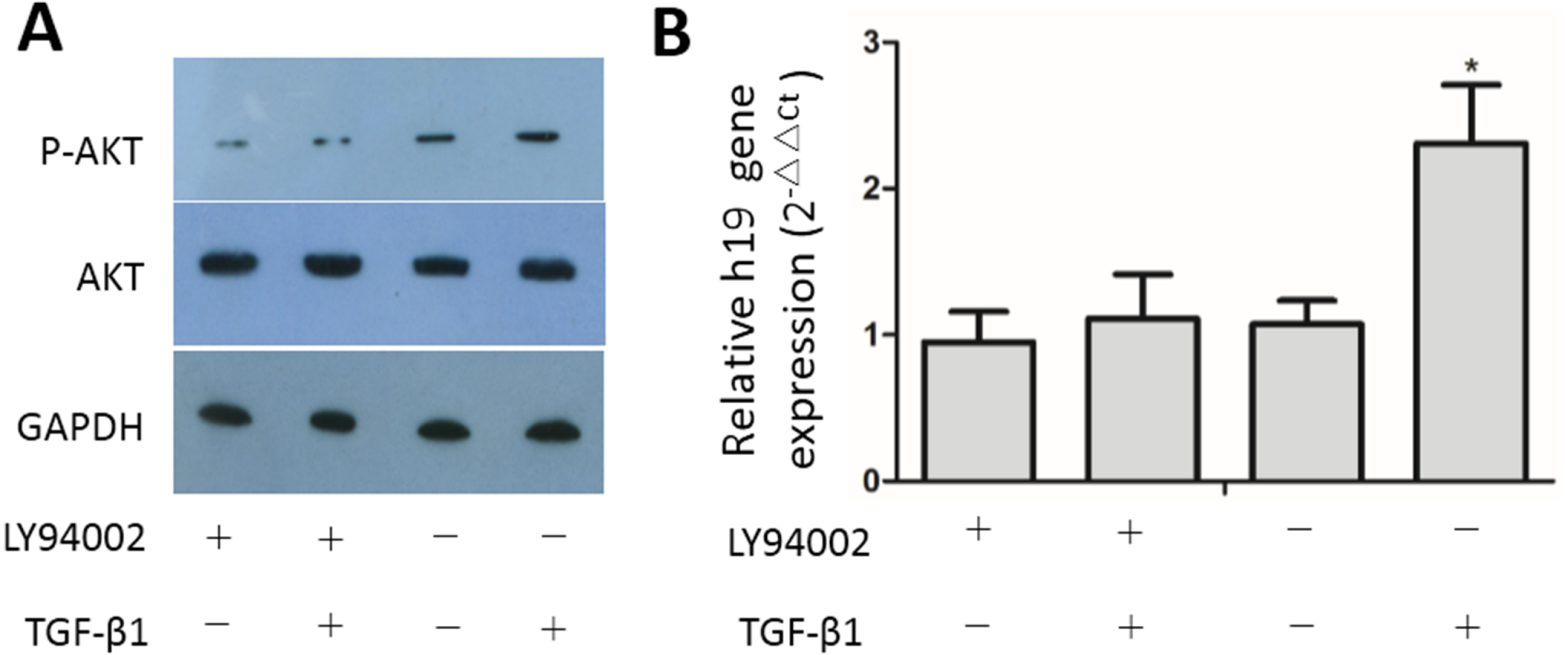
Inhibition of the PI3K/AKT pathway suppressed activation of TGF-β1-induced upregulation of H19 expression. Confluent MAC-T cells were serum-starved for 24 h and pretreated with the inhibitor at 20 µM in serum-free media for 30 min, and then the cells were treated with TGF-β1 at 2 ng/mL for 36 h. (A) Phosphorylated Akt and total Akt expression in cells was analyzed by western blotting. (B) Expression of H19 in cells was analyzed by RT-qPCR. GAPDH was used as an internal control. ^∗^*p* < 0.05 vs control.

### H19-mediated TGF-*β*1-induced EMT and ECM protein expression in MAC-T cells

To investigate whether H19 mediates TGF-β1-induced EMT in MAC-T cells, we established MAC-T cell clones stably overexpressing H19 by introducing the lentiviral vector of CD513B-1 containing the H19 sequence (Fig. 4A). High-level GFP-expression was detected by direct observation with a fluorescent microscope in MAC-T cells transfected with both the CD513B-1 and CD513B-1-H19 vectors after selection with puromycin (Fig. 4B). Overexpression of H19 in MAC-T cells was further confirmed by RT-qPCR (Fig. 4C). Western blotting results showed that overexpression of H19 significantly promoted TGF-β1-induced EMT in MAC-T cells compared to that in CD513B-1 control vector-transfected cells, as indicated by increased α-SMA, vimentin and N-cadherin expression and decreased E-cadherin, β-catenin and pan-cytokeratin expression (Fig. 4D). In addition, overexpression of H19 promoted TGF-β1-induced ECM expression increases in albumin, collagen 1, fibronectin and MMP9 (Fig. 4D).

### TGF-β1-stimulated H19 requires AKT activation

A previous publication linked H19 expression to the phosphatidylinositol-3-kinase/protein kinase B (PI3K/AKT) pathway in response to TGF-β1 treatment (Matouk et al., 2014). Here, we found that TGF-β1 treatment activated AKT protein in MAC-T cells, and this activation was effectively inhibited by LY 294002, a specific chemical inhibitor of the PI3K/AKT pathway (Fig. 5A). Furthermore, inhibiting activation of the PI3K/AKT pathway by LY294002 abolished TGF-β1-induced up-regulation of H19 in MAC-T cells (Fig. 5B). These data suggest that TGF-β1-induced EMT in MAC-T cells is mediated by the TGF-β1/AKT/H19 axis.

### Identification of H19 promoter region

**Figure 6:**
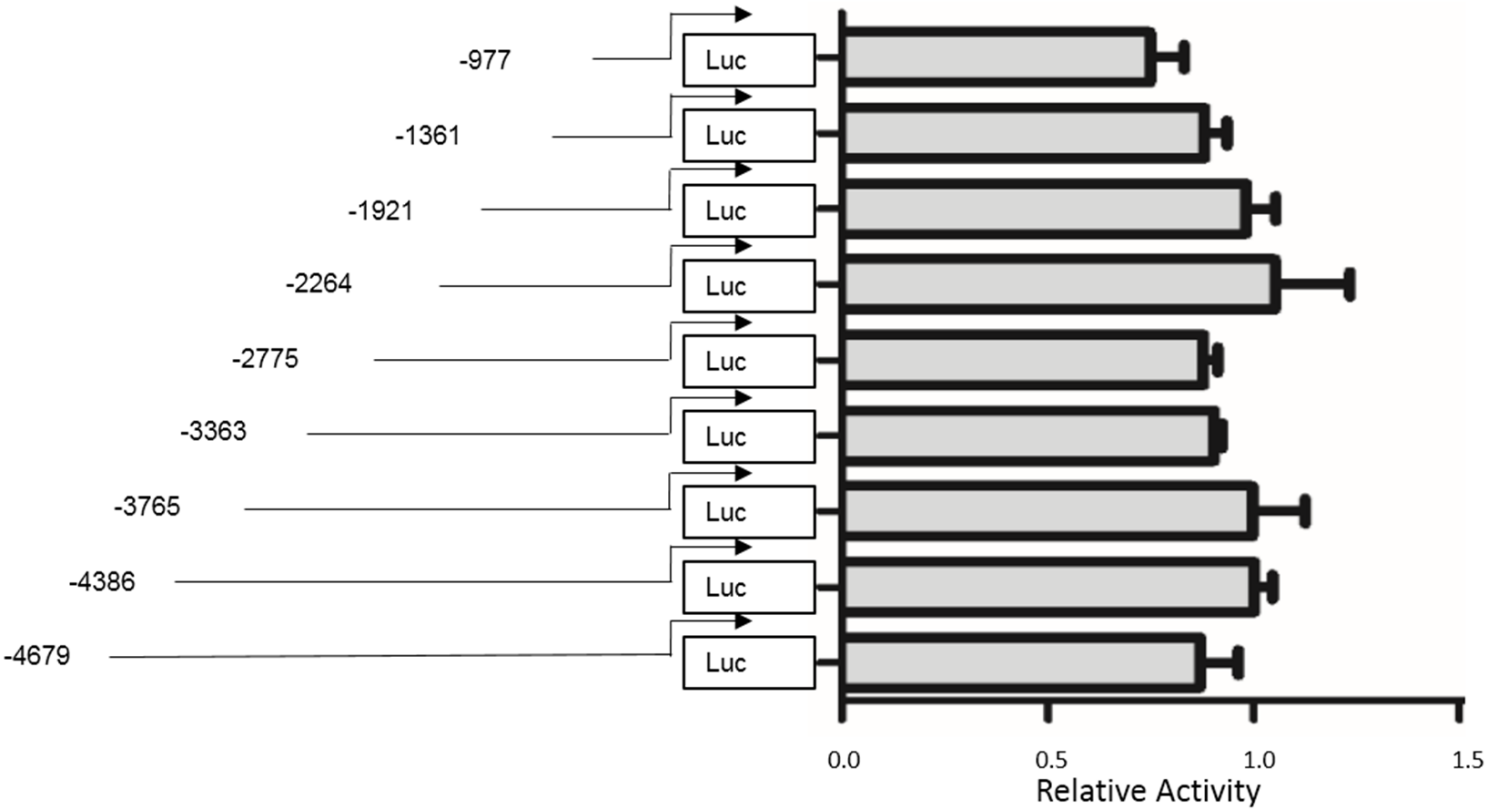
Functional analysis of different lengths of putative promoter sequences of H19 in MAC-T cells. The different 5′ truncated fragments upstream of H19 gene sequences were generated and inserted into the Luc-vector. Fragment lengths are indicated relative to the transcription start site, and the activity of the fragments constructs relative to a promoter-less construct is shown. *P* > 0.05 among all groups.

To identify the promoter region of H19 gene which responds to the TGF-β1 treatment, luciferase reporter plasmids were constructed by inserting truncations of a 4,679-base pair upstream segment of H19. All constructs were confirmed by DNA sequencing. Luciferase activities were tested by luciferase assay in the MAC-T cell line. We did not detect the H19 promoter region within the upstream 4,976 base pair sequences of H19 responsible for TGF-β1-induced EMT in MAC-T cells. As shown in Fig. 6, transcriptional activity did not differ among all examined segments.

## Discussion

It has been reported that H19 upregulation is linked to the EMT process in carcinogenesis and embryogenesis, and induction of EMT by different approaches (e.g., hypoxia, TGF-β) in cancer cells is accompanied by H19 upregulation (Oviedo-Boyso et al., 2007). EMT is considered a potential source of change in myofibroblasts in tissue fibrosis (Zou et al., 2017), and bovine mastitis typically causes varying degrees of fibrosis in mammary glands. We hypothesized that H19 upregulation is involved in mammary fibrosis induced by mastitis.

Here, we found that exposure of MAC-T cells to TGF-β1 stimulation induced EMT mediated by H19 upregulation, representing another source of fibroblasts involved in mammary gland fibrosis in bovine.

We first observed that the expression level of H19 was upregulated in both mastitic mammary tissue compared to normal tissue, indicating that H19 is involved in the immune response of mammary glands during bovine mastitis. During bacterial invasion, mammary epithelial cells play an important role in inducing a relevant innate immune response in mammary glands by immunological factor release (Zhang et al., 2016). H19 upregulation was also observed in inflammatory MAC-T cells induced by the gram-negative and gram-positive bacterial cell wall components LPS and LTA, suggesting that H19 is involved in the immune response process of epithelial cells triggered by pathogens.

Following inflammation, some types of cells produce TGF-β1, and excess TGF-β1 contributes to a pathologic excess of tissue fibrosis (Branton & Kopp, 1999). EMT plays an important role in repair and scar formation following epithelial injury. Evidence that TGF-β1 induces EMT in alveolar epithelial cells *in vitro* and *in vivo* suggests that alveolar epithelial cells serve as a source of myofibroblasts in lung fibrosis (Willis & Borok, 2007). In this study, we found that TGF-β1 expression was upregulated in MAC-T cells under LPS or LTA stimulation. To investigate whether excess TGF-β1 affected MAC-T cells in an autocrine manner, thus leading to EMT, we treated MAC-T cells with exogenous TGF-β1. TGF-β1-induced protein expression changes in several well-known EMT makers, such as downregulation of E-cadherin and upregulation of α-SMA and N-cadherin, in MAC-T cells, indicating that TGF-β1-induced EMT had occurred. Myofibroblasts release a variety of excessive extracellular matrix proteins contributing to organ fibrosis (Hinz et al., 2007). We found that TGF-β1 stimulated increased expression of extracellular matrix proteins including collagen type 1, MMP9, and albumin in MAC-T cells.

Interestingly, TGF-β1 stimulated the expression of H19 in MAC-T cells. We next established an epithelial cell line stably overexpressing H19 to assess the relationship between H19 and TGF-β1-induced EMT in MAC-T cells. We found that H19 overexpression enhanced TGF-β1-induced EMT in MAC-T cells, indicating that high H19 expression was associated with EMT, which is consistent with the previous observation that TGF-β1 is associated with EMT and inflammation (Franco et al., 2010; Gal et al., 2008; Salgado et al., 2017).

Accumulating studies have shown that AKT is an important regulator of EMT in most cell types (Larue & Bellacosa, 2005; Lee & Han, 2010; Maseki et al., 2012). In our study, we found that TGF-β1 activated the PI3K/AKT signal pathway in MAC-T cells, and a specific inhibitor of the PI3K/AKT pathway inhibited AKT activation. Additionally, this inhibitor abated TGF-β1-induced upregulation of H19 in MAC-T cells, suggesting that increased H19 expression in MAC-T cells was induced by TGF-β1 through the PI3K/AKT pathway. This finding is consistent with the results of a previous study linking H19 expression to the PI3K/AKT pathway in response to TGF-β1 treatment (Matouk et al., 2014). Finally, we attempted to identify the promoter region responsive to TGF-β1 treatment by using a luciferase reporter assay to detect the upstream 4.679-kb sequence of H19 gene, but no promoter responsive to TGF-β1 was found within the examined sequence regions. Further studies are needed to determine the mechanism by which TGF-β1 regulate the expression of H19 in MAC-T cells.

## Conclusions

In summary, we found that H19 is highly expressed in both bovine mastitic tissue and inflammatory bovine epithelial cells, and lncRNA H19 is involved in TGF-β1-induced EMT and ECM protein synthesis in bovine epithelial cells through the PI3K/AKT signaling pathway. In addition, our results clearly demonstrate the molecular mechanism of H19 as responsive to TGF-β1 and that EMT occurs *in vivo* during bovine mastitis. Based on our results, mammary epithelial cells may be a source of myofibroblasts in inflamed mammary glands, thereby contributing to mammary gland fibrosis.

##  Supplemental Information

10.7717/peerj.3950/supp-1 File S1 A list of primers for PCR.

10.7717/peerj.3950/supp-2 Data S1 Full-length uncropped blots for all figures Raw data of full-length uncropped blots.

10.7717/peerj.3950/supp-3 Data S2 Raw data for all analysis

## Additional Information and Declarations

### Data Availability

The following information was supplied regarding data availability: The raw data is contained in the Supplemental Files.
