# Supplementary material for: lncRNA H19 is involved in TGF-β1-induced epithelial to mesenchymal transition in bovine epithelial cells through PI3K/AKT Signaling Pathway
Source: PeerJ. 2017 Oct 17;5:e3950. doi: 10.7717/peerj.3950 (PMC5649593; doi:10.7717/peerj.3950)
Supplement: File S1 — A list of primers for PCR. [file peerj-05-3950-s001.pdf]

Supplemental file 1: A list of primers for PCR

Gene	Forward primer	Reverse primer	Cycles
H19	CGCCACTTCACCCACTGTA	CGCTGCCTTCTCTCTCATTC	40
TNF-α	GTTCTCCCCATGACACCACCTG	GGGAGAAGAGAGTCAGACAGGC	40
TGF-β1	TTACAACAGTACCCGCGACC	AGAGAGCAACACAGGTTCGG	40
-977bp—-140bp	GGGGTACCCCTCCTAAGGTGCCAAGTCG	CCCAAGCTTCAACCACGCAGCCCTATTT	35
-1361bp—-140bp	GGGGTACCGACAGAACACTCGGGACAATG	CCCAAGCTTCAACCACGCAGCCCTATTT	35
-1921bp—-140bp	GGGGTACCCCACGCAAGACCTTGGAG	CCCAAGCTTCAACCACGCAGCCCTATTT	35
-2264bp—-140bp	GGGGTACCGCTCTAAAGCCTGGACTCTGTG	CCCAAGCTTCAACCACGCAGCCCTATTT	35
-2775bp—-140bp	GGGGTACCAAGCCTGTCCGAGCTACCC	CCCAAGCTTCAACCACGCAGCCCTATTT	35
-3363bp—-140bp	GGGGTACCGGCATCAGTGAGCAGAAACA	CCCAAGCTTCAACCACGCAGCCCTATTT	35
-3765bp—-140bp	GGGGTACCGCGGTGGTGATGGAGACA	CCCAAGCTTCAACCACGCAGCCCTATTT	35
-4386bp—-140bp	GGGGTACCCTCAGTCCCGAACCCTCC	CCCAAGCTTCAACCACGCAGCCCTATTT	35
-4679bp—-140bp	GGGGTACCCACAGTGAGAAGGGAGCAAGG	CCCAAGCTTCAACCACGCAGCCCTATTT	35
